# What's Important Now (WIN) Fall Prevention Protocol for Geriatric Patients Following Total Hip Replacement

**DOI:** 10.7759/cureus.113939

**Published:** 2026-08-04

**Authors:** Manoj Agnihotri, Ajit Dabholkar

**Affiliations:** 1 Musculoskeletal Physiotherapy, Babandada Patil College of Physiotherapy, DY Patil Deemed to be University, Navi Mumbai, IND; 2 Physiotherapy, DY Patil Deemed to be University, Navi Mumbai, IND

**Keywords:** geriatrics, orthopedics, physical medicine, rehabilitation, total hip replacement

## Abstract

Falls are one of the most frequently reported postoperative complications among geriatric patients after total hip replacement (THR) surgery and may lead to fractures, extended hospital stays, delayed rehabilitation, loss of functional independence, and decreased quality of life. Various physiological changes associated with age, muscle weakness from surgery, altered gait patterns, polypharmacy, and environmental hazards all contribute to fall risk during recovery. The What's Important Now (WIN) Fall Prevention Protocol (FPP) is a multidisciplinary, evidence-based rehabilitation program designed to reduce fall risk and maximize postoperative outcomes for older adults who are undergoing THR. The protocol incorporates progressive strength and balance exercises, gait re-education, patient and caregiver education, environmental modifications, medication review, and psychological support into a structured six-week rehabilitation program. A progression of exercises (range of motion, Theraband resistance training, functional strengthening, balance exercises, and task-oriented gait exercises) is outlined to support mobility and confidence while ensuring safe recovery. The protocol also focuses on personalized evaluation and customized treatment, which helps ensure that patients receive the most appropriate care for their needs and that they are more likely to stick to their rehabilitation program. The current evidence for each component of the protocol is summarized, emphasizing the need for a holistic, patient-centered approach to fall prevention in older people. The WIN Fall Prevention Protocol incorporates tried and tested rehabilitation principles and effective implementation strategies to give clinicians a structured framework for better postoperative safety, fall-related complications, and functional recovery after THR. Additional prospective clinical trials are needed to assess the effectiveness of the protocol and validate its effects on patient outcomes in different healthcare environments.

## Introduction

Total hip replacement (THR) is a common and serious complication in geriatric patients with falls [1]. Fall risk is heightened during the period of time following major surgery, and in older people, this risk is compounded by diminished muscle mass, bone density, balance, and visual acuity [2]. These changes, along with the restrictions from surgery, increase the risk for fall fracture, extended hospital stay, and reduced quality of life [3]. Therefore, the development of effective fall prevention methods is an important element in achieving good outcomes in geriatric patients receiving THR.

THR is one of the most frequently performed orthopedic procedures in elderly patients and is typically performed for osteoarthritis, hip fracture, and other forms of degenerative hip disease [4]. It is effective and safe in providing pain relief and mobility, but there is an inherent risk of falling during the early postoperative period. After surgery, patients often experience decreased mobility, muscle weakness, and changes in gait [5]. A fall at this time can result in long-term rehabilitation, permanent disability, or even death, and prevention is an essential part of post-surgical care.

There are many causes for falls in this population. The majority of THR patients are discharged equipped with assistive devices such as walkers or crutches, which many older adults have trouble dealing with [6]. Restricted mobility and discomfort following surgery further hinder balance and coordination. Proprioception, which is the ability to sense the position of the body in space, declines with age, leaving limited awareness of body position, and especially after surgery is performed, a factor that is important to the postural control system [7]. These sensory and physical impairments, coupled with environmental risk factors (slippery surfaces, insufficient lighting, and furniture placement), significantly increase the risk of falls.

A customized, multi-component fall prevention protocol (FPP) is thus required to reduce these risks and to foster recovery [8]. The best programs involve more than just physical therapy but also environmental modifications, patient/caregiver education, and monitoring during rehabilitation. Some core components encompass exercise strengthening, balance training, correct use of assistive devices, pain control, and home modification. Physiotherapists have a key role in helping to strengthen and restore mobility and provide information on safe walking and transferring. The review of medication is also important, as analgesics and chronic medications may cause dizziness, sedation, or hypotension and should be regularly reviewed for their effect on balance and cognition [9].

This comprehensive approach is used in the What's Important Now (WIN) Fall Prevention Program for geriatric patients after THR. It employs evidence-based methods to improve physical function, offer psychological support, and make the space safe [10]. The patient-centered evaluation sets the stage for a tailored prevention plan that addresses each patient's specific risk factors, and structured education ensures patient and caregiver safety at home post-discharge. The program will use this multifactorial and patient-centered approach to reduce the incidence of falls, improve postoperative care, maximize recovery, minimize long-term disability, and improve safety and quality of life in the postoperative period.

## Technical report

The WIN Fall Prevention Protocol is a research-based program to prevent falls and minimize fall risk in the older adult patient following a total hip replacement (THR) surgery. Geriatric patients are at risk because of their age, and numerous factors affect their recovery after THR surgery, which can increase their risk for falling [11]. Thus, fall risk identification and fall risk reduction are essential for achieving optimal outcomes. This fall reduction protocol is holistic and comprehensive, promoting recovery of geriatric THR patients from both a physical and environmental perspective.

Fall risk factors in geriatric THR patients

The first step of the WIN Fall Prevention Protocol is to recognize that in all geriatric patients, there is a natural risk of falling after undergoing THR surgery. The most frequently performed THR surgeries are performed due to osteoarthritis, hip fractures, and other degenerative diseases; however, there are also postoperative problems that are experienced in many patients, such as loss of muscle strength, loss of balance, and impaired mobility [12]. The WIN protocol understands that the patient's postoperative difficulties, along with the natural aging process, create a much higher risk of fall events and the risk for subsequent fall-associated complications. Complications from falls include fractures, extended hospitalizations, and decreased quality of life. Therefore, fall prevention should be a priority area in patient care for patients after THR [13]. Falls in elderly people are multifactorial and complex. Falls are caused by both internal and external factors, including muscle weakness, loss of proprioception, and poor lighting and slippery surfaces. The WIN Fall Prevention Protocol acknowledges that there are many factors involved with falls in the geriatric population, and its intent is to decrease the risk for falls through the use of internal and external factors. Hence, the WIN protocol is a holistic and person-centered approach to fall prevention [14].

Pre-implementation instructions: WIN exercise protocol

Before initiating the WIN exercise protocol, the patient and caregiver should receive comprehensive education regarding the purpose of the exercises, correct exercise techniques, and the importance of performing them safely and consistently according to the prescribed program. They should also be informed about warning signs indicating that the exercises should be discontinued immediately, including increased hip pain, sudden swelling, redness or discharge from the surgical wound, dizziness, chest pain, sudden worsening of breathlessness, or loss of balance, and advised to seek prompt medical attention if any of these symptoms occur. The exercise environment should be made safe by removing potential trip hazards, ensuring adequate lighting, providing access to stable support when required, and encouraging the use of appropriate footwear to minimize the risk of falls. In addition, psychological and emotional support should be provided through reassurance, positive encouragement, and active caregiver supervision to reduce fear of falling, improve confidence, promote safe participation, and enhance adherence to the exercise program.

Table 1 presents a detailed fall prevention exercise protocol for geriatric patients following total hip replacement (THR) surgery. The protocol spans three distinct phases: the initial 1-2 weeks, the mid-phase of 2-4 weeks, and the later 4-6 weeks post-surgery. Each phase includes exercises focused on warming up, walking, strength, balance, and cooling down, all designed to progressively build strength, stability, and confidence while reducing the risk of falls.

**Table 1:** Progressive Fall Prevention Exercise Protocol for Geriatric Patients Post-THR THR: total hip replacement, ROM: range of motion

Phase	Frequency and Duration	Components	Exercise Protocol
Weeks 1-2	3 days/week; 30-40 minutes/session; 2 sets × 10 repetitions; 2-3 minutes rest between sets	Warm-up (5 minutes); walking (5-10 minutes); strength (5-10 minutes); balance (5-10 minutes); cool-down (5 minutes)	Warm-up: active ROM exercises for cervical spine, upper limb, and lower limb; walking: walk 5 m × 10 repetitions on a level surface; strength: yellow TheraBand knee extension and flexion (10 repetitions each), half squats (10 repetitions, 10-second hold); balance: single-leg standing (5-second hold), standing hip abduction (10 repetitions, 5-second hold); cool-down: self-stretching of wrist flexors/extensors, triceps, ankle dorsiflexors/plantar flexors, and breathing exercises
Weeks 2-4	3 days/week; 30-40 minutes/session; 2 sets × 10 repetitions; 2-3 minutes rest between sets	Warm-up (5 minutes); walking (5-10 minutes); strength (5-10 minutes); balance (5-10 minutes); cool-down (5 minutes)	Warm-up: active ROM exercises for cervical spine, upper limb, and lower limb; walking: stair climbing (10 repetitions), tandem walking for 5 m × 10 repetitions; strength: red TheraBand knee extension and flexion (10 repetitions each), half squats (10 repetitions, 15-second hold); balance: single-leg standing (10-second hold × 10), standing hip abduction and hip extension (10 repetitions, 10-second hold); cool-down: self-stretching and breathing exercises
Weeks 4-6	3 days/week; 30-40 minutes/session; 2 sets × 10 repetitions; 2-3 minutes rest between sets	Warm-up (5 minutes); walking (5-10 minutes); strength (5-10 minutes); balance (5-10 minutes); cool-down (5 minutes)	Warm-up: active ROM exercises for cervical spine, upper limb, and lower limb; walking: figure-of-eight walking (10 repetitions), walking while counting numbers for 5 m × 10 repetitions; strength: green TheraBand knee extension and flexion (10 repetitions each), half squats (10 repetitions, 20-second hold), reverse lunges with weights (10 repetitions, 10-second hold); balance: single-leg standing on medium-density foam (5-second hold), standing hip abduction and hip extension on medium-density foam (10 repetitions, 5-second hold); cool-down: self-stretching and breathing exercises

WIN Fall Prevention Protocol components

The multi-faceted approach is the key strength of the WIN Fall Prevention Protocol. The WIN protocol includes multiple components that collectively offer a complete program to minimize the fall risk in geriatric patients receiving THR.

Exercise and Physical Therapy

Strengthening exercises, balance training, and mobility support are crucial to the protocol. The purpose of the exercise component of the protocol is to help the patient regain or improve his or her physical function, which is sometimes impaired after THR. The WIN protocol details the types of exercises to be performed, such as walking, Theraband strength training exercises, and single-leg standing balance exercises. It increases the intensity of the exercises during the protocol, which starts with basic mobility and strengthening exercises to more complex balance exercises in the last few weeks [15].

Patient/Educator and Caregiver Education

Patient/caregiver education is a key component of the WIN protocol. The educational component aims to educate the patient about fall risk factors and help him/her learn how to reduce fall risk through the use of assistive devices and through knowledge of environmental hazards. The educational component also educates the patient and caregiver about when and how to ask for help and how their home can be made safer [16].

Environmental Modifications

The WIN protocol suggests changes to the patient's external environment to minimize the external risk factors that can increase the risk of falls. These are all recommended changes, such as sufficient lighting, eliminating obstacles, and using assistive devices (such as walkers or crutches) [17].

Medication Management

Medications given following surgery, such as pain management medications and those used for chronic medical conditions, can have side effects that increase the risk for falling (such as dizziness or sedation). This is why it is important to regularly review and adapt medications to reduce this risk, a practice supported by the WIN protocol [18].

Psychological and Emotional Support

The WIN protocol also covers the psychological and emotional issues facing the patient, including the anxiety that comes with another fall, and not feeling able to manage physical tasks. The WIN protocol takes physical and emotional aspects of the patient's health into account, so that the patient can be physically and emotionally ready to manage his or her postoperative care [19]. The WIN Fall Prevention Protocol allows for flexibility to address each patient's individual needs. This is done through a personalized evaluation of the patient's physical function, home environment, and psychological state to create a personalized fall prevention program.

In the WIN protocol, there is also continuous monitoring for improvement to determine if the patient is improving and to identify any emerging fall risk factors as soon as possible [20]. An individualized approach to implementation is required due to the fact that each geriatric patient will have a different recovery period after the THR, depending on their previous medical condition, comorbid conditions, and family and friend support. The WIN protocol is flexible enough to be tailored to address the individual needs of each patient, thus enhancing the likelihood that the patient will be able to perform the fall prevention strategies in the WIN protocol and safely complete their postoperative rehabilitation [21].

## Discussion

The WIN Fall Prevention Protocol offers a clear, evidence-based protocol to minimize the risk of falls in elderly patients post-total hip replacement (THR). The protocol incorporates many strategies, such as the use of exercise, education, modification of the environment, and psychological support, to address the complex causes of fall risk. The WIN protocol aligns and builds on the key findings of most studies, which showed the need for a patient-centered and holistic fall prevention strategy for the elderly.

The impact of physical factors, particularly muscle weakness and balance issues, on fall risk following THR was clearly illustrated by Hunter et al. (2020) [22]. A year after surgery, the authors found that a significant number of patients had continued muscle weakness, which was a significant factor in their falls. In keeping with these results, the WIN protocol places a high emphasis on strengthening and balance exercises designed to improve mobility and decrease the likelihood of falls in patients after THR surgery. The focus of the structured exercise plan outlined in the WIN protocol on walking, strength, and balance training offers a structured approach to addressing physical deficits reported by Hunter et al. (2020) [22] linked to higher fall risk.

Likewise, Walston (2012) [23] highlighted the role of sarcopenia, or skeletal muscle wasting associated with ageing, as one of the most important risk factors for falls among older people and suggested that sarcopenia plays an important role in post-surgical recovery and fall prevention. The WIN protocol's strength and balance exercises are designed to overcome muscle weakness and facilitate recovery. The exercises in the protocol also follow a progression, building up as the patient's recovery advances, as described in that study. In addition, exercises targeted for strength development, such as TheraBand exercises and squatting, are also a feature of the protocol that can help prevent sarcopenia, thereby making it more relevant for fall prevention.

The study conducted by Vaishya and Vaish (2020) [24] examined the relationship between age, falls after surgery, and falls in older adults. They explored the relationships between age and fall risk after THR, particularly in those having difficulty with gait and balance. The WIN protocol directly addresses these concerns by including exercises that help improve gait, such as walking on different surfaces and using assistive devices such as walkers and crutches. Hence, the objective of enhancing mobility and balance as set forth in the WIN protocol is in line with the recommendations of Vaishya and Vaish (2020) on rehabilitation to minimize falls.

Orts-Cortés et al. (2024) [25] studied the effectiveness of nurse-led interventions to prevent falls in older adults. Their research confirmed the need for education as well as physical rehabilitation to decrease fall risk. In line with these results, the WIN Fall Prevention Protocol has an educational component where patients and caregivers are educated about fall prevention strategies and appropriate assistive devices. Therefore, the WIN protocol places emphasis on the need for a multi-faceted approach in fall prevention, including education for the patient.

The WIN protocol is similar to the abovementioned studies but has the benefit of being holistic and patient-specific. A unique physical and environmental risk assessment is conducted for each patient to determine specific factors that increase fall risk. The individualized nature of the WIN protocol increases the overall success of the intervention, as the studies mentioned above provided generalized fall prevention plans.

## Conclusions

Finally, the WIN Fall Prevention Protocol successfully combines the best practices of previous fall prevention studies and tailors them to an individualized, multi-dimensional approach. The WIN protocol is a holistic and patient-centered approach to reducing falls and enhancing the outcomes of elderly patients after THR surgery by addressing the physical, environmental, and psychological factors that contribute to falls. The protocol is worthy of further prospective clinical trials to assess its effectiveness and demonstrate its impact on patient outcomes in a variety of healthcare environments.
